# Supplementary material for: Identification of Y-chromosome Turnover in Newts Fails to Support a Sex Chromosome Origin for the Triturus Balanced Lethal System
Source: Genome Biol Evol. 2025 Aug 14;17(9):evaf155. doi: 10.1093/gbe/evaf155 (PMC12410432; doi:10.1093/gbe/evaf155)
Supplement: evaf155_Supplementary_Data [file evaf155_supplementary_data.pdf]

Supplementary Data

Identification of Y-chromosome turnover in newts fails to support a sex chromosome origin for the *Triturus* balanced lethal system

James France, Wiesław Babik, Milena Cvijanović, Katarzyna Dude, Ana Ivanović, Tijana Vučić, Ben Wielstra

Table of Contents:

**Figure S1: Screening in *T. ivanbureschi***	Page 2
**Figure S2: Screening in *T. cristatus* and *T. macedonicus***	Page 2
**Figure S3: Screening in other *Triturus* species**	Page 3
**Figure S4: PCR validation of TiY-384959-short**	Page 4
**Figure S5: PCR validation of TiY-137941-long**	Page 5
**Table S1: Sample information**	Page 6
**Table S2: Linkage map statistics**	Page 10
**Table S3: Primer sequences**	Page 10

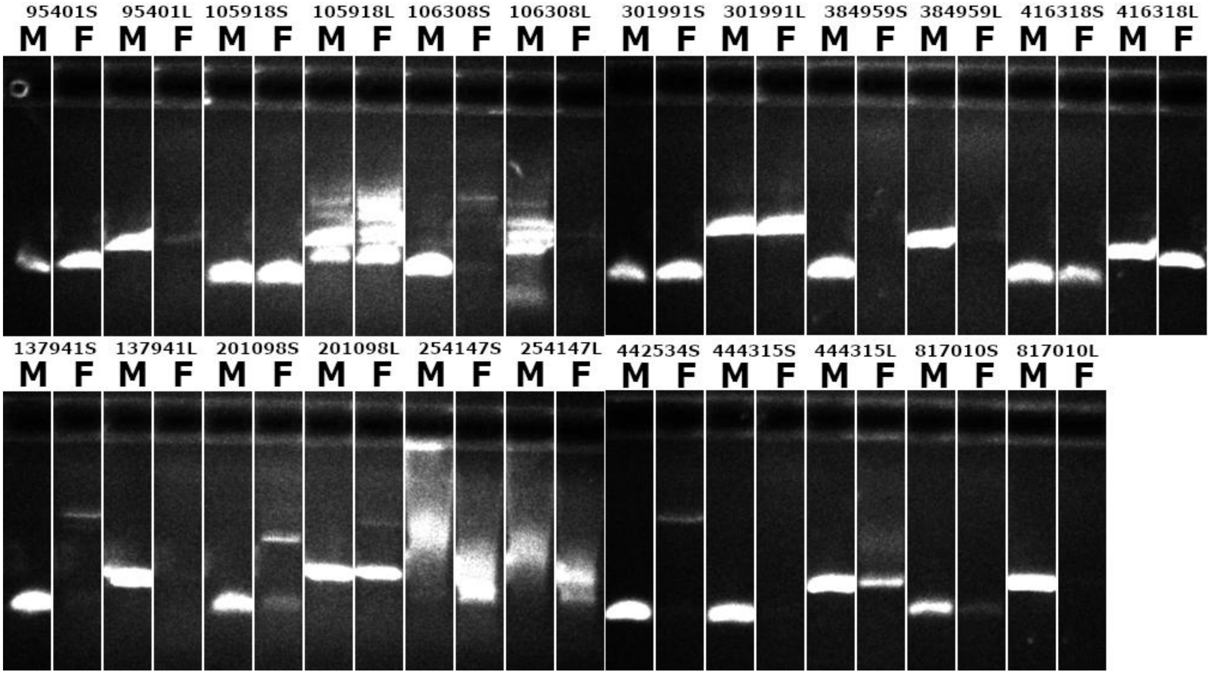

Figure S1: PCR screening of 23 primer pairs designed for candidate Y-linked markers for sex specific amplification in *Triturus ivanbureschi*. Label M indicates the male sample and label F indicates female. Markers are indicated by number followed by either S (for primer pairs designed for the short product – c.a. 100 bp) or L (for primer pairs designed for the long product – c.a. 200 bp). 12 pairs show male specific amplification.

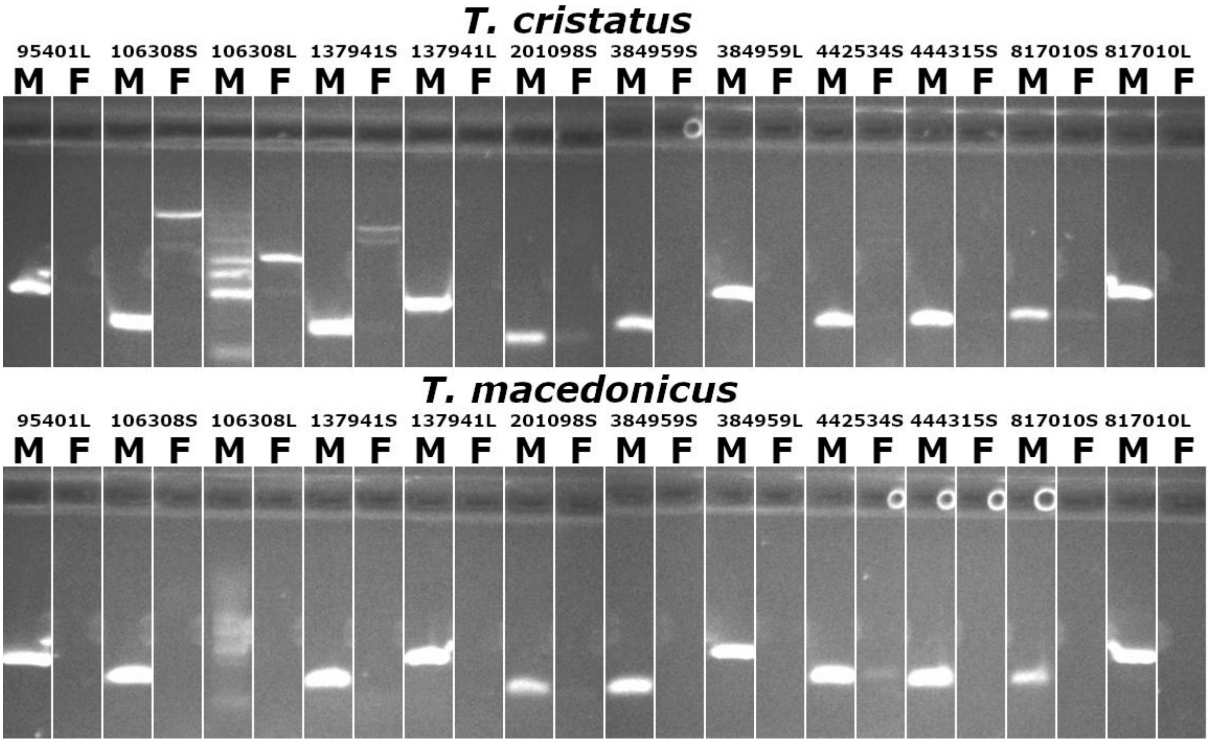

Figure S2: Further PCR screening of 12 primer pairs for Y-linked markers (that show male specific amplification in *T. ivanbureschi*) in *T. cristatus* and *T. macedonicus*. Label M indicates the male sample and label F indicates female. Markers are indicated by number followed by either S (for primer pairs designed for the short product – c.a. 100 bp) or L (for primer pairs designed for the long product – c.a. 200 bp). 6 primer pairs show strong amplification in males of both species with no product at all visible in females (several other show varying degrees of weak amplification in females).

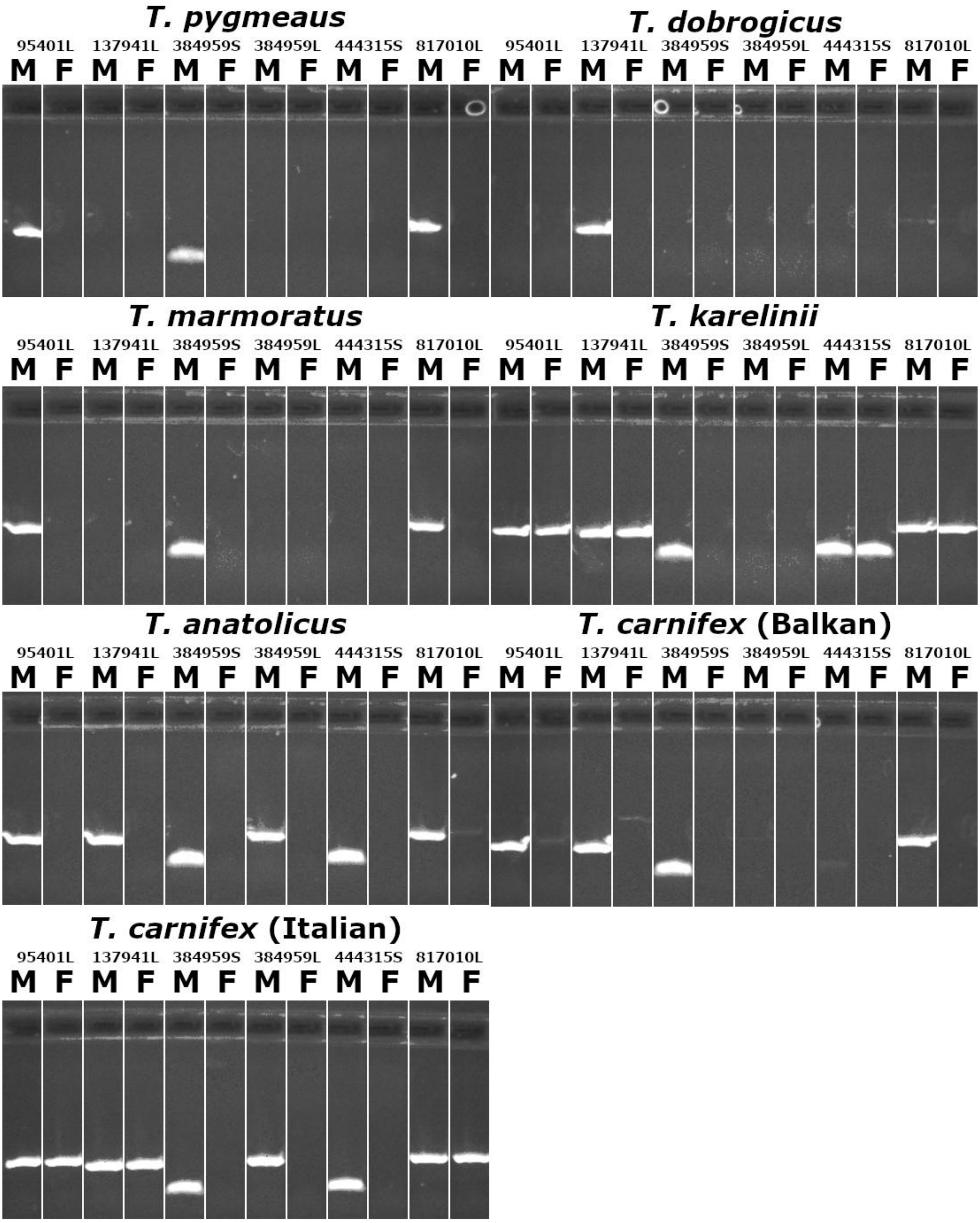

Figure S3: PCR screening of 6 primer pairs for Y-linked markers (that show confirmed male specific amplification in three *Triturus* species) in all other *Triturus* species (except for *T. rudolfi*, which was not yet described at the time of study). Label M indicates the male sample and label F indicates female. Markers are indicated by number followed by either S (for primer pairs designed for the short product – c.a. 100 bp) or L (for primer pairs designed for the long product – c.a. 200 bp). No marker shows male specific amplification in all species, however TiY-384959-short is successful in all other than *T. dobrogicus*. The only marker to show any amplification in *T. dobrogicus* is TiY-137941-long.

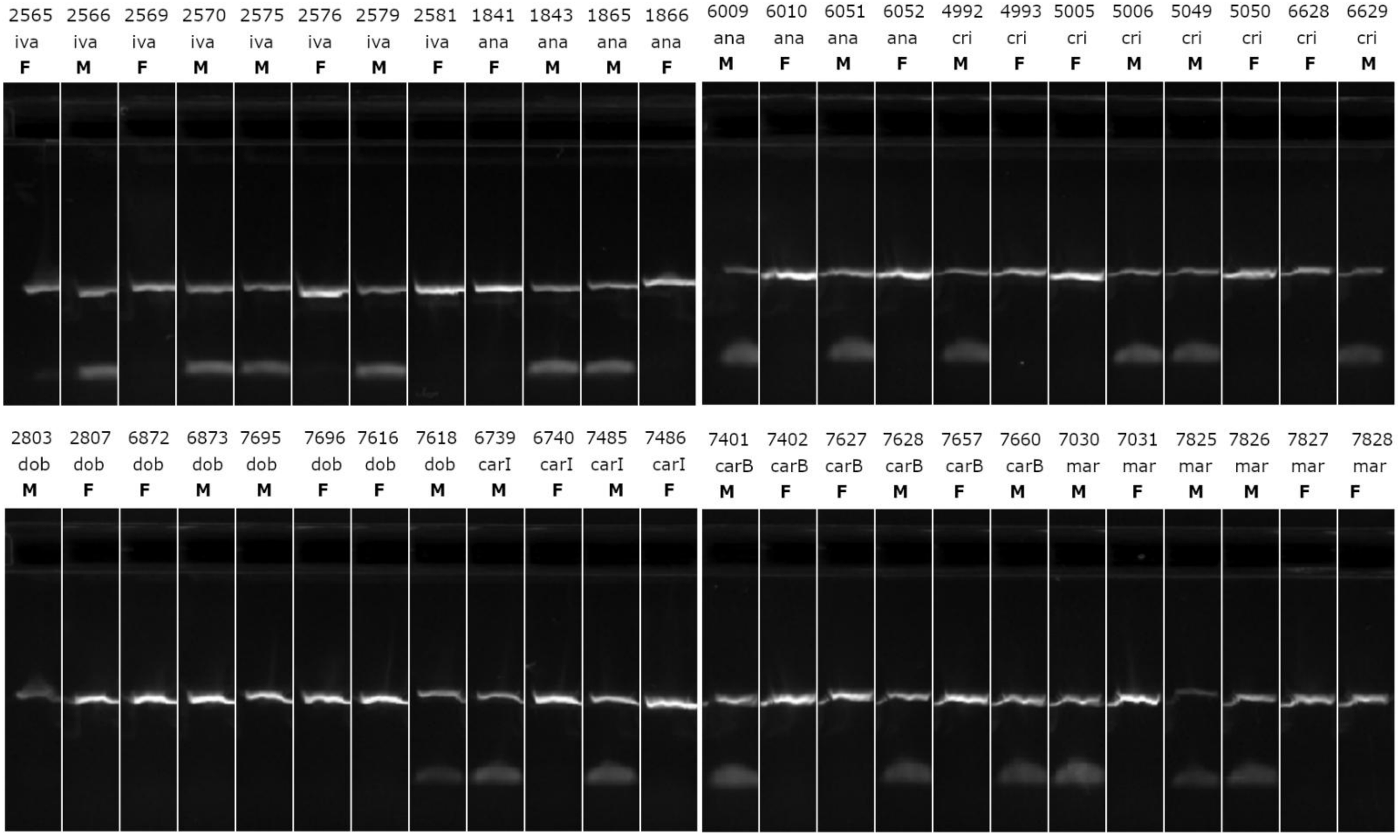

Figure S4: PCR validation of Y-linked marker TiY-384959-short (lower band) in combination with control marker CDK-17 (upper band) across 48 samples from six *Triturus* species. Abbreviations as follows: *T. ivanbureschi* – iva, *T. anatolicus* - ana, *T. cristatus* – cri, *T. dobrogicus* – dob, *T. carnifex* – car (I indicates Italian lineage, B indicates Balkan), *T. marmoratus* – mar. Label M indicates the male sample and label F indicates female. In accordance with the results from screening in male/female pairs TiY-384959-short amplifies male specifically in all species except *T. dobrogicus* (where it failed to amplify at all expect in a single male).

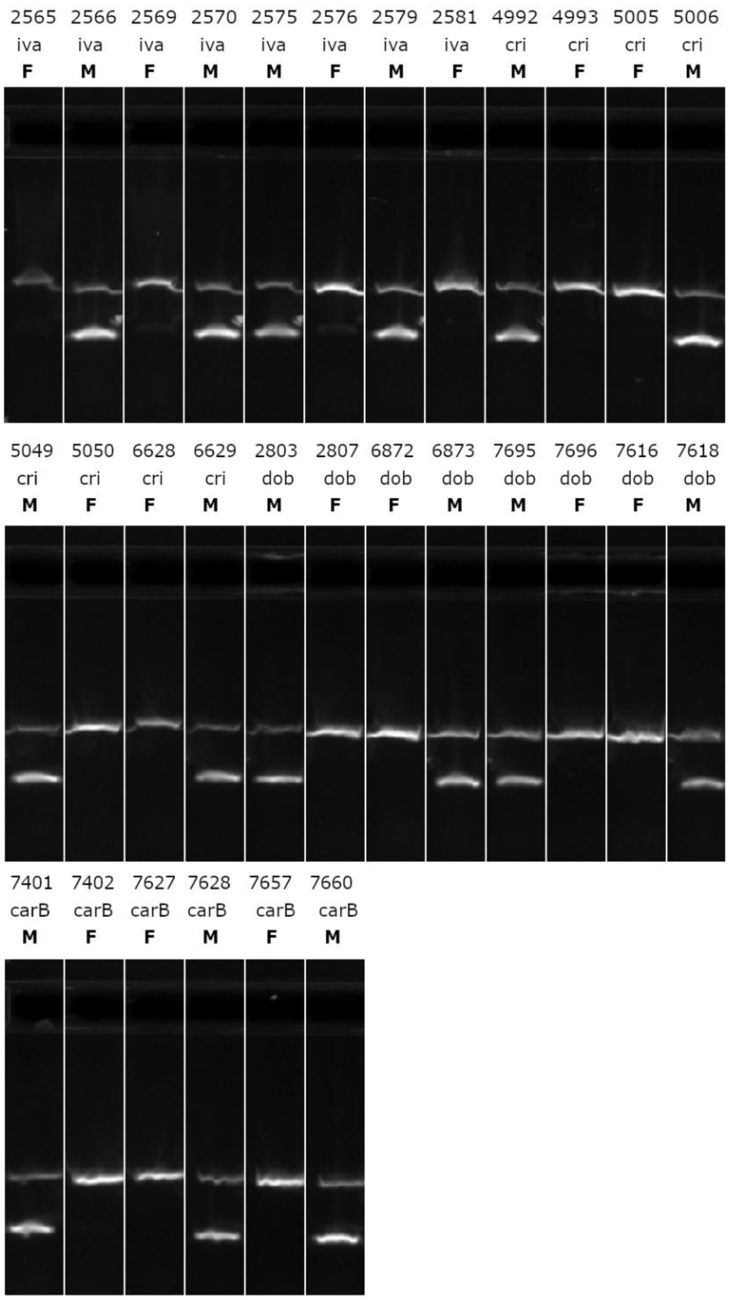

Figure S5: PCR validation of Y-linked marker TiY-137941-long (lower band) in combination with control marker CDK-17 (upper band) across 30 samples from four *Triturus* species that naturally occur within the Balkan region. Abbreviations as follows: *T. ivanbureschi* – iva, *T. cristatus* – cri, *T. dobrogicus* – dob, *T. carnifex* – car (B indicates Balkan lineage). Label M indicates the male sample and label F indicates female. In accordance with the results from screening in male/female pairs TiY-137941-long amplifies male specifically in all species including *T. dobrogicus*.

Table S1: Samples used for RADseq and initial PCR screening during this study. All samples designated for use as 'Sexed RADseq' and 'Map parent' were collected in Zli Dol (Pčinja district, Serbia, 42°25 N; 22°27 E). Samples designated as validation were previously reported in Wielstra et al. (2017a) and Wielstra et al. (2017b). All other samples were captive bred. RADseq data from all *Triturus ivanbureschi* samples is available as part of NCBI bioproject PRJNA1173742 (France et al. 2024b).

Sample	Species	Sex	Use
**BW_0008**	*T. ivanbureschi*	Male	Map parent
**BW_0009**	*T. ivanbureschi*	Female	Map parent
**BW_0798**	*T. cristatus*	Male	Screening
**BW_0799**	*T. cristatus*	Female	Screening
**BW_0803**	*T. macedonicus*	Male	Screening
**BW_0804**	*T. macedonicus*	Female	Screening
**BW_0786**	*T. Pygmaeus*	Male	Screening
**BW_0787**	*T. Pygmaeus*	Female	Screening
**BW_0788**	*T. Dobrogicus*	Male	Screening
**BW_0789**	*T. Dobrogicus*	Female	Screening
**BW_0790**	*T. Marmoratus*	Male	Screening
**BW_0791**	*T. Marmoratus*	Female	Screening
**BW_0792**	*T. Karelinii*	Male	Screening
**BW_0793**	*T. Karelinii*	Female	Screening
**BW_0796**	*T. Anatolicus*	Male	Screening
**BW_0797**	*T. Anatolicus*	Female	Screening
**BW_0800**	*T. Carnifex (B)*	Male	Screening
**BW_0801**	*T. Carnifex (B)*	Female	Screening
**BW_0805**	*T. Carnifex (I)*	Male	Screening
**BW_0806**	*T. Carnifex (I)*	Female	Screening
**2565**	*T. ivanbureschi*	Female	Validation
**2566**	*T. ivanbureschi*	Male	Validation
**2569**	*T. ivanbureschi*	Female	Validation
**2570**	*T. ivanbureschi*	Male	Validation
**2575**	*T. ivanbureschi*	Male	Validation
**2576**	*T. ivanbureschi*	Female	Validation
**2579**	*T. ivanbureschi*	Male	Validation
**2581**	*T. ivanbureschi*	Female	Validation
**1841**	*T. ivanbureschi*	Female	Validation
**1843**	*T. ivanbureschi*	Male	Validation
**1865**	*T. ivanbureschi*	Male	Validation
**1866**	*T. anatolicus*	Female	Validation
**6009**	*T. anatolicus*	Male	Validation
**6010**	*T. anatolicus*	Female	Validation
**6051**	*T. anatolicus*	Male	Validation
**6052**	*T. anatolicus*	Female	Validation
**4992**	*T. cristatus*	Male	Validation
**4993**	*T. cristatus*	Female	Validation
**5005**	*T. cristatus*	Female	Validation
**5006**	*T. cristatus*	Male	Validation
**5049**	*T. cristatus*	Male	Validation

Sample	Species	Sex	Use
BW_0551	*T. ivanbureschi*	Unknown	Map offspring
BW_0552	*T. ivanbureschi*	Unknown	Map offspring
BW_0553	*T. ivanbureschi*	Unknown	Map offspring
BW_0554	*T. ivanbureschi*	Unknown	Map offspring
BW_0555	*T. ivanbureschi*	Unknown	Map offspring
BW_0556	*T. ivanbureschi*	Unknown	Map offspring
BW_0557	*T. ivanbureschi*	Unknown	Map offspring
BW_0558	*T. ivanbureschi*	Unknown	Map offspring
BW_0559	*T. ivanbureschi*	Unknown	Map offspring
BW_0560	*T. ivanbureschi*	Unknown	Map offspring
BW_0561	*T. ivanbureschi*	Unknown	Map offspring
BW_0562	*T. ivanbureschi*	Unknown	Map offspring
BW_0563	*T. ivanbureschi*	Unknown	Map offspring
BW_0564	*T. ivanbureschi*	Unknown	Map offspring
BW_0565	*T. ivanbureschi*	Unknown	Map offspring
BW_0566	*T. ivanbureschi*	Unknown	Map offspring
BW_0567	*T. ivanbureschi*	Unknown	Map offspring
BW_0568	*T. ivanbureschi*	Unknown	Map offspring
BW_0569	*T. ivanbureschi*	Unknown	Map offspring
BW_0570	*T. ivanbureschi*	Unknown	Map offspring
BW_0571	*T. ivanbureschi*	Unknown	Map offspring
BW_0572	*T. ivanbureschi*	Unknown	Map offspring
BW_0573	*T. ivanbureschi*	Unknown	Map offspring
BW_0574	*T. ivanbureschi*	Unknown	Map offspring
BW_0575	*T. ivanbureschi*	Unknown	Map offspring
**BW_0576**	*T. ivanbureschi*	Unknown	Map offspring
**BW_0577**	*T. ivanbureschi*	Unknown	Map offspring
**BW_0578**	*T. ivanbureschi*	Unknown	Map offspring
**BW_0579**	*T. ivanbureschi*	Unknown	Map offspring
**BW_0580**	*T. ivanbureschi*	Unknown	Map offspring
**BW_0581**	*T. ivanbureschi*	Unknown	Map offspring
**BW_0585**	*T. ivanbureschi*	Unknown	Map offspring
**BW_0587**	*T. ivanbureschi*	Unknown	Map offspring
**BW_0588**	*T. ivanbureschi*	Unknown	Map offspring
**BW_0590**	*T. ivanbureschi*	Unknown	Map offspring
**BW_0592**	*T. ivanbureschi*	Unknown	Map offspring
**BW_0594**	*T. ivanbureschi*	Unknown	Map offspring
**BW_0595**	*T. ivanbureschi*	Unknown	Map offspring
**BW_0597**	*T. ivanbureschi*	Unknown	Map offspring
**BW_0599**	*T. ivanbureschi*	Unknown	Map offspring
**BW_0601**	*T. ivanbureschi*	Unknown	Map offspring

Sample	Species	Sex	Use
**5050**	*T. cristatus*	Female	Validation
**6628**	*T. cristatus*	Female	Validation
**6629**	*T. cristatus*	Male	Validation
**2565**	*T. ivanbureschi*	Female	Validation
**2803**	*T. dobrogicus*	Male	Validation
**2807**	*T. dobrogicus*	Female	Validation
**6872**	*T. dobrogicus*	Female	Validation
**6873**	*T. dobrogicus*	Male	Validation
**7695**	*T. dobrogicus*	Male	Validation
**7696**	*T. dobrogicus*	Female	Validation
**7616**	*T. dobrogicus*	Female	Validation
**7618**	*T. dobrogicus*	Male	Validation
**6739**	*T. carnifex (I)*	Male	Validation
**6740**	*T. carnifex (I)*	Female	Validation
**7485**	*T. carnifex (I)*	Male	Validation
**7486**	*T. carnifex (I)*	Female	Validation
**7401**	*T. carnifex (B)*	Male	Validation
**7402**	*T. carnifex (B)*	Female	Validation
**7627**	*T. carnifex (B)*	Female	Validation
**7628**	*T. carnifex (B)*	Male	Validation
**7657**	*T. carnifex (B)*	Female	Validation
**7660**	*T. carnifex (B)*	Male	Validation
**7030**	*T. marmoratus*	Male	Validation
**7031**	*T. marmoratus*	Female	Validation
**7825**	*T. marmoratus*	Male	Validation
**7826**	*T. marmoratus*	Male	Validation
**7827**	*T. marmoratus*	Female	Validation
**7828**	*T. marmoratus*	Female	Validation
**2803**	*T. dobrogicus*	Male	Validation
**BW_0758**	*T. ivanbureschi*	Male	Sexed RADseq
**BW_0759**	*T. ivanbureschi*	Male	Sexed RADseq
**BW_0761**	*T. ivanbureschi*	Male	Sexed RADseq
**BW_0762**	*T. ivanbureschi*	Male	Sexed RADseq
**BW_0763**	*T. ivanbureschi*	Male	Sexed RADseq
**BW_0764**	*T. ivanbureschi*	Male	Sexed RADseq
**BW_0765**	*T. ivanbureschi*	Male	Sexed RADseq
**BW_0766**	*T. ivanbureschi*	Male	Sexed RADseq
**BW_0767**	*T. ivanbureschi*	Male	Sexed RADseq
**BW_0768**	*T. ivanbureschi*	Male	Sexed RADseq
**BW_0769**	*T. ivanbureschi*	Male	Sexed RADseq
**BW_0770**	*T. ivanbureschi*	Male	Sexed RADseq
**BW_0771**	*T. ivanbureschi*	Male	Sexed RADseq
**BW_0772**	*T. ivanbureschi*	Female	Sexed RADseq
**BW_0773**	*T. ivanbureschi*	Female	Sexed RADseq
**BW_0774**	*T. ivanbureschi*	Female	Sexed RADseq
**BW_0775**	*T. ivanbureschi*	Female	Sexed RADseq

Sample	Species	Sex	Use
**BW_0602**	*T. ivanbureschi*	Unknown	Map offspring
**BW_0605**	*T. ivanbureschi*	Unknown	Map offspring
**BW_0606**	*T. ivanbureschi*	Unknown	Map offspring
**BW_0607**	*T. ivanbureschi*	Unknown	Map offspring
**BW_0609**	*T. ivanbureschi*	Unknown	Map offspring
**BW_0611**	*T. ivanbureschi*	Unknown	Map offspring
**BW_0612**	*T. ivanbureschi*	Unknown	Map offspring
**BW_0615**	*T. ivanbureschi*	Unknown	Map offspring
**BW_0617**	*T. ivanbureschi*	Unknown	Map offspring
**BW_0618**	*T. ivanbureschi*	Unknown	Map offspring
**BW_0619**	*T. ivanbureschi*	Unknown	Map offspring
**BW_0620**	*T. ivanbureschi*	Unknown	Map offspring
**BW_0621**	*T. ivanbureschi*	Unknown	Map offspring
**BW_0623**	*T. ivanbureschi*	Unknown	Map offspring
**BW_0625**	*T. ivanbureschi*	Unknown	Map offspring
**BW_0626**	*T. ivanbureschi*	Unknown	Map offspring
**BW_0628**	*T. ivanbureschi*	Unknown	Map offspring
**BW_0631**	*T. ivanbureschi*	Unknown	Map offspring
**BW_0633**	*T. ivanbureschi*	Unknown	Map offspring
**BW_0634**	*T. ivanbureschi*	Unknown	Map offspring
**BW_0635**	*T. ivanbureschi*	Unknown	Map offspring
**BW_0636**	*T. ivanbureschi*	Unknown	Map offspring
**BW_0639**	*T. ivanbureschi*	Unknown	Map offspring
**BW_0644**	*T. ivanbureschi*	Unknown	Map offspring
**BW_0645**	*T. ivanbureschi*	Unknown	Map offspring
**BW_0646**	*T. ivanbureschi*	Unknown	Map offspring
**BW_0648**	*T. ivanbureschi*	Unknown	Map offspring
**BW_0650**	*T. ivanbureschi*	Unknown	Map offspring
**BW_0653**	*T. ivanbureschi*	Unknown	Map offspring
**BW_0655**	*T. ivanbureschi*	Unknown	Map offspring
**BW_0657**	*T. ivanbureschi*	Unknown	Map offspring
**BW_0659**	*T. ivanbureschi*	Unknown	Map offspring
**BW_0660**	*T. ivanbureschi*	Unknown	Map offspring
**BW_0661**	*T. ivanbureschi*	Unknown	Map offspring
**BW_0664**	*T. ivanbureschi*	Unknown	Map offspring
**BW_0665**	*T. ivanbureschi*	Unknown	Map offspring
**BW_0666**	*T. ivanbureschi*	Unknown	Map offspring
**BW_0667**	*T. ivanbureschi*	Unknown	Map offspring
**BW_0668**	*T. ivanbureschi*	Unknown	Map offspring
**BW_0670**	*T. ivanbureschi*	Unknown	Map offspring
**BW_0671**	*T. ivanbureschi*	Unknown	Map offspring
**BW_0673**	*T. ivanbureschi*	Unknown	Map offspring
**BW_0674**	*T. ivanbureschi*	Unknown	Map offspring
**BW_0676**	*T. ivanbureschi*	Unknown	Map offspring
**BW_0677**	*T. ivanbureschi*	Unknown	Map offspring
**BW_0678**	*T. ivanbureschi*	Unknown	Map offspring

Sample	Species	Sex	Use
**BW_0776**	*T. ivanbureschi*	Female	Sexed RADseq
**BW_0777**	*T. ivanbureschi*	Female	Sexed RADseq
**BW_0778**	*T. ivanbureschi*	Female	Sexed RADseq
**BW_0780**	*T. ivanbureschi*	Female	Sexed RADseq
**BW_0781**	*T. ivanbureschi*	Female	Sexed RADseq
**BW_0782**	*T. ivanbureschi*	Female	Sexed RADseq
**BW_0783**	*T. ivanbureschi*	Female	Sexed RADseq
**BW_0784**	*T. ivanbureschi*	Female	Sexed RADseq
**BW_0785**	*T. ivanbureschi*	Female	Sexed RADseq
**BW_2095**	*T. ivanbureschi*	Male	Sexed RADseq
**BW_2096**	*T. ivanbureschi*	Male	Sexed RADseq
**BW_2097**	*T. ivanbureschi*	Male	Sexed RADseq
**BW_2098**	*T. ivanbureschi*	Male	Sexed RADseq
**BW_2099**	*T. ivanbureschi*	Male	Sexed RADseq
**BW_2100**	*T. ivanbureschi*	Male	Sexed RADseq
**BW_2101**	*T. ivanbureschi*	Male	Sexed RADseq
**BW_2102**	*T. ivanbureschi*	Male	Sexed RADseq
**BW_2103**	*T. ivanbureschi*	Male	Sexed RADseq
**BW_2104**	*T. ivanbureschi*	Male	Sexed RADseq
**BW_2105**	*T. ivanbureschi*	Male	Sexed RADseq
**BW_2106**	*T. ivanbureschi*	Male	Sexed RADseq
**BW_2107**	*T. ivanbureschi*	Male	Sexed RADseq
**BW_2108**	*T. ivanbureschi*	Male	Sexed RADseq
**BW_2110**	*T. ivanbureschi*	Male	Sexed RADseq
**BW_2111**	*T. ivanbureschi*	Female	Sexed RADseq
**BW_2112**	*T. ivanbureschi*	Female	Sexed RADseq
**BW_2113**	*T. ivanbureschi*	Female	Sexed RADseq
**BW_2114**	*T. ivanbureschi*	Female	Sexed RADseq
**BW_2115**	*T. ivanbureschi*	Female	Sexed RADseq
**BW_2116**	*T. ivanbureschi*	Female	Sexed RADseq
**BW_2117**	*T. ivanbureschi*	Female	Sexed RADseq
**BW_2118**	*T. ivanbureschi*	Female	Sexed RADseq
**BW_2119**	*T. ivanbureschi*	Female	Sexed RADseq
**BW_2120**	*T. ivanbureschi*	Female	Sexed RADseq
**BW_2121**	*T. ivanbureschi*	Female	Sexed RADseq
**BW_2122**	*T. ivanbureschi*	Female	Sexed RADseq
**BW_2123**	*T. ivanbureschi*	Female	Sexed RADseq
**BW_2124**	*T. ivanbureschi*	Female	Sexed RADseq
**BW_2125**	*T. ivanbureschi*	Female	Sexed RADseq
**BW_2126**	*T. ivanbureschi*	Female	Sexed RADseq
**BW_2127**	*T. ivanbureschi*	Male	Sexed RADseq
**BW_0536**	*T. ivanbureschi*	Unknown	Map offspring
**BW_0537**	*T. ivanbureschi*	Unknown	Map offspring
**BW_0538**	*T. ivanbureschi*	Unknown	Map offspring
**BW_0539**	*T. ivanbureschi*	Unknown	Map offspring
**BW_0540**	*T. ivanbureschi*	Unknown	Map offspring

Sample	Species	Sex	Use
**BW_0679**	*T. ivanbureschi*	Unknown	Map offspring
**BW_0680**	*T. ivanbureschi*	Unknown	Map offspring
**BW_0682**	*T. ivanbureschi*	Unknown	Map offspring
**BW_0683**	*T. ivanbureschi*	Unknown	Map offspring
**BW_0684**	*T. ivanbureschi*	Unknown	Map offspring
**BW_0685**	*T. ivanbureschi*	Unknown	Map offspring
**BW_0686**	*T. ivanbureschi*	Unknown	Map offspring
**BW_0689**	*T. ivanbureschi*	Unknown	Map offspring
**BW_0690**	*T. ivanbureschi*	Unknown	Map offspring
**BW_0691**	*T. ivanbureschi*	Unknown	Map offspring
**BW_0692**	*T. ivanbureschi*	Unknown	Map offspring
**BW_0693**	*T. ivanbureschi*	Unknown	Map offspring
**BW_0694**	*T. ivanbureschi*	Unknown	Map offspring
**BW_0695**	*T. ivanbureschi*	Unknown	Map offspring
**BW_0697**	*T. ivanbureschi*	Unknown	Map offspring
**BW_0698**	*T. ivanbureschi*	Unknown	Map offspring
**BW_0699**	*T. ivanbureschi*	Unknown	Map offspring
**BW_0701**	*T. ivanbureschi*	Unknown	Map offspring
**BW_0702**	*T. ivanbureschi*	Unknown	Map offspring
**BW_0703**	*T. ivanbureschi*	Unknown	Map offspring
**BW_0704**	*T. ivanbureschi*	Unknown	Map offspring
**BW_0705**	*T. ivanbureschi*	Unknown	Map offspring
**BW_0706**	*T. ivanbureschi*	Unknown	Map offspring
**BW_0707**	*T. ivanbureschi*	Unknown	Map offspring
**BW_0708**	*T. ivanbureschi*	Unknown	Map offspring
**BW_0709**	*T. ivanbureschi*	Unknown	Map offspring
**BW_0710**	*T. ivanbureschi*	Unknown	Map offspring
**BW_0711**	*T. ivanbureschi*	Unknown	Map offspring
**BW_0712**	*T. ivanbureschi*	Unknown	Map offspring
**BW_0713**	*T. ivanbureschi*	Unknown	Map offspring
**BW_0715**	*T. ivanbureschi*	Unknown	Map offspring
**BW_0717**	*T. ivanbureschi*	Unknown	Map offspring
**BW_0719**	*T. ivanbureschi*	Unknown	Map offspring
**BW_0720**	*T. ivanbureschi*	Unknown	Map offspring
**BW_0722**	*T. ivanbureschi*	Unknown	Map offspring
**BW_0724**	*T. ivanbureschi*	Unknown	Map offspring
**BW_0725**	*T. ivanbureschi*	Unknown	Map offspring
**BW_0726**	*T. ivanbureschi*	Unknown	Map offspring
**BW_0727**	*T. ivanbureschi*	Unknown	Map offspring
**BW_0728**	*T. ivanbureschi*	Unknown	Map offspring
**BW_0733**	*T. ivanbureschi*	Unknown	Map offspring
**BW_0735**	*T. ivanbureschi*	Unknown	Map offspring
**BW_0737**	*T. ivanbureschi*	Unknown	Map offspring
**BW_0740**	*T. ivanbureschi*	Unknown	Map offspring
**BW_0741**	*T. ivanbureschi*	Unknown	Map offspring
**BW_0744**	*T. ivanbureschi*	Unknown	Map offspring

Sample	Species	Sex	Use
**BW_0541**	*T. ivanbureschi*	Unknown	Map offspring
**BW_0542**	*T. ivanbureschi*	Unknown	Map offspring
**BW_0543**	*T. ivanbureschi*	Unknown	Map offspring
**BW_0544**	*T. ivanbureschi*	Unknown	Map offspring
**BW_0545**	*T. ivanbureschi*	Unknown	Map offspring
**BW_0546**	*T. ivanbureschi*	Unknown	Map offspring
**BW_0547**	*T. ivanbureschi*	Unknown	Map offspring
**BW_0548**	*T. ivanbureschi*	Unknown	Map offspring
**BW_0549**	*T. ivanbureschi*	Unknown	Map offspring
**BW_0550**	*T. ivanbureschi*	Unknown	Map offspring

Sample	Species	Sex	Use
**BW_0745**	*T. ivanbureschi*	Unknown	Map offspring
**BW_0746**	*T. ivanbureschi*	Unknown	Map offspring
**BW_0748**	*T. ivanbureschi*	Unknown	Map offspring
**BW_0749**	*T. ivanbureschi*	Unknown	Map offspring
**BW_0750**	*T. ivanbureschi*	Unknown	Map offspring
**BW_0751**	*T. ivanbureschi*	Unknown	Map offspring
**BW_0752**	*T. ivanbureschi*	Unknown	Map offspring
**BW_0753**	*T. ivanbureschi*	Unknown	Map offspring
**BW_0754**	*T. ivanbureschi*	Unknown	Map offspring
**BW_0755**	*T. ivanbureschi*	Unknown	Map offspring

Group	Number of Markers	Length (cM)
1	391	105.6
2	931	102.1
3	1005	98.8
4	764	98.4
5	331	93.8
6	427	93.3
7	381	91.7
8	600	91.4
9	710	91.4
10	903	86.4
11	314	84.3
12	476	82.3
**Total**	**7233**	**1119.6**

Table S2: Characteristics of linkage groups within the linkage map constructed based on RADseq data from 160 *T. ivanbureschi* samples from a full-sibling family.

Primer Pair	Forward Primer Sequence	Reverse Primer Sequence	Product Length (bp)
**CDK-17**	GGCATGGGAAGAACAGAAGA	CCATCTGCTTGGACTGTTGA	537
**TiY-444315-short**	AGTTCGAGCCAGTACTTTTAGC	CAAACACACGAAAGCACAGTG	111
**TiY-444315-long**	CACTGTGCTTTCGTGTGTTTG	TGTACTAGAAAGGGTGGGGG	>105
**TiY-137941-short**	GTCACAGCAGCAAATGGTCC	CAGAAGAAGGGCATCTGGG	104
**TiY-137941-long**	GTCACAGCAGCAAATGGTCC	CCTCTGCTCTGCCTTCACAG	>166
**TiY-95401-short**	CTAGATTCCGGTGAGGCAGG	GGCCCATAGCACCAACATTC	137
**TiY-95401-long**	GCGTACGGAGTGATTATCCCC	ACACTGCTGCGGAACTGAAG	>199
**TiY-105918-short**	TGAGGATCTGGCTCAATCGC	TCTCCAAAGGTAACGCGCTG	82
**TiY-105918-long**	AATCTTGTCCACCAGTGTGC	AATTCAGCAGCCCACATGCC	>185
**TiY-442534-short**	AGGGGCATAAGTGGAGGGAC	AGGGTCTGAAAAGGGCCATC	114
**TiY-416318-short**	TGGGTTTCCAAGTCTCCTCAG	ACTTTCAAGAGTAAGGAGCAGAAG	89
**TiY-416318-long**	TGGGTTTCCAAGTCTCCTCAG	TGGAGGCCTGAAGTAATAAGCC	>166
**TiY-254147-short**	CCGGTCACATCTCCTTCGAG	GACTGGGCTTGAGAGTCTCG	150
**TiY-254147-long**	CCGGTCACATCTCCTTCGAG	TCGAAGCAGATGTGACTGGG	>163
**TiY-384959-short**	TGCAGCACAGCAGTAGACTC	CCTTCTCGCATGGACCCTAC	92
**TiY-384959-long**	GTAGGGTCCATGCGAGAAGG	AGGTGTCGTGTGCCTACTTC	>168
**TiY-201098-short**	TAAACCAGCAAAGCCACCAC	TGTACAATTCCTGCGTAACCG	83
**TiY-201098-long**	CCACCCCCAAGCACTTAAAG	TGTGTGGGTCCCAAAAGTGG	>200
**TiY-817010-short**	TCTGCTTTGTGTCTGAAGCTTG	TGTGTGTTCCTGTTGGGCTG	127
**TiY-817010-long**	TCACCTACCACCACAGTTGC	CACTCCTGACTATGGGCCTG	>186
**TiY-301991-short**	GGGGAGTCAGGGTTGTCATG	TCTACTAGCTCACAGGGCAC	87
**TiY-301991-long**	GGGGAGTCAGGGTTGTCATG	TGGGGTTTCCTACTCAGCTG	>194
**TiY-106308-short**	AGCAAGTTCCAGGAGCTTCC	AGAGCACATGAAGGACCAGC	125
**TiY-106308-long**	TCACCAGCAGAGTTTCTCCG	TGAAGGACCAGTGGATGCTG	>186

Table S3: Sequences of all primers used in this study, CDK-17 is an autosomal marker used as a control, all others are candidate Y-linked markers developed for *T. ivanbureschi*.
